# Development of the EMAP tool facilitating existential communication between general practitioners and cancer patients

**DOI:** 10.1080/13814788.2017.1326479

**Published:** 2017-08-11

**Authors:** Elisabeth Assing Hvidt, Dorte Gilså Hansen, Jette Ammentorp, Lars Bjerrum, Søren Cold, Pål Gulbrandsen, Frede Olesen, Susanne S. Pedersen, Jens Søndergaard, Connie Timmermann, Helle Timm, Niels Christian Hvidt

**Affiliations:** a Department of Public Health, Research Unit of General Practice, Faculty of Health Sciences, University of Southern Denmark, Odense, Denmark;; b Institute of Regional Health Research, Faculty of Health Sciences, University of Southern Denmark, Odense, Denmark;; c Health Services Research Unit, Lillebaelt Hospital, Vejle, Denmark;; d Department of Public Health, Section of General Practice and Research Unit for General Practice, University of Copenhagen, Copenhagen, Denmark;; e Department of Oncology, Medical Faculty, Odense University Hospital, University of Southern Denmark, Odense, Denmark;; f Department of Social Medicine, Institute of Clinical Medicine, University of Oslo, Oslo, Norway;; g HØKH Research Centre, Akershus University Hospital, Lorenskog, Norway;; h Department of Public Health, The Research Unit for General Practice, Aarhus University, Aarhus, Denmark;; i Department of Psychology, Unit of Medical Psychology, Faculty of Health Sciences, University of Southern Denmark, Odense, Denmark;; j Department of Cardiology, Odense University Hospital, Odense, Denmark;; k Research Unit of General Practice, University of Southern Denmark, Odense, Denmark;; l The Danish Knowledge Centre for Rehabilitation and Palliative Care (REHPA), National Institute of Public Health, University of Southern Denmark, Odense, Denmark

**Keywords:** General practice/family medicine, quality of care, communication, patient involvement, palliative and terminal care

## Abstract

**Background:** General practice recognizes the existential dimension as an integral part of multidimensional patient care alongside the physical, psychological and social dimensions. However, general practitioners (GPs) report substantial barriers related to communication with patients about existential concerns.

**Objectives:** To describe the development of the EMAP tool facilitating communication about existential problems and resources between GPs and patients with cancer.

**Methods:** A mixed-methods design was chosen comprising a literature search, focus group interviews with GPs and patients (*n* = 55) and a two-round Delphi procedure initiated by an expert meeting with 14 experts from Denmark and Norway.

**Results:** The development procedure resulted in a semi-structured tool containing suggestions for 10 main questions and 13 sub-questions grouped into four themes covering the existential dimension. The tool utilized the acronym and mnemonic EMAP (existential communication in general practice) indicating the intention of the tool: to provide a map of possible existential problems and resources that the GP and the patient can discuss to find points of reorientation in the patient’s situation.

**Conclusion:** This study resulted in a question tool that can serve as inspiration and help GPs when communicating with cancer patients about existential problems and resources. This tool may qualify GPs’ assessment of existential distress, increase the patient’s existential well-being and help deepen the GP–patient relationship.

Key messages

- A communication tool (the EMAP tool) has been made available to lessen GP-reported barriers to communication with patients about existential issues.
- The effectiveness of the EMAP tool in a European general practice setting must be evaluated.

## Introduction

General practice recognizes the existential dimension as an integral part of multidimensional patient care alongside the physical, psychological and social dimensions [1]. This recognition is supported by comprehensive evidence demonstrating that patients facing serious illness frequently experience multiple existential problems and concerns that have a negative impact on their physical and psychological health [2]. Furthermore, studies show that patients wish to have their existential concerns and resources addressed as part of their medical care [3–5].

In the medical literature, the existential dimension includes issues related to identity, personal integrity, an unfulfilled past, as well as issues relating to concerns about the present and the future such as meaninglessness, hopelessness, death, futility and spiritual/religious concerns [6]. In this research context, we understand the existential dimension potentially, but not mandatorily, to involve spiritual and religious factors [7].

Addressing the existential dimension is, however, hindered by substantial self-reported barriers among GPs including lack of confidence in the language and the concepts involved, lack of communication training and skill, fear of causing discomfort to the patients and lack of spiritual/religious self-awareness [8,9].

In an attempt to lessen these reported barriers, several so-called spiritual history taking tools have been designed internationally [10]. Evaluation studies have shown that physicians benefit from using such tools during the medical encounter in that they offer an element of structure and support when addressing existential issues [10]. Although existing tools derived primarily from an American healthcare context frame existential support broadly with the religiously neutral concept of spirituality, they nonetheless reflect societies in which religion plays a different role than in secular Europe marked by a crisis of religious beliefs and institutions [11,12]. Contextual differences and variations are emphasized in two European studies in which the American-developed FICA tool (faith, importance, community, address) has been sought implemented in clinical care in Flanders, Belgium and in Germany [13,14]. These studies also show that although considered helpful when discussing a patient’s spirituality, the physicians recommend that tools be developed that are adapted to the local society, taking cultural and religious variations into account (focusing less on religion and spirituality) and that phrase questions in spoken language [13,14].

The aim of this paper is to describe the development of such a context-adequate tool that facilitates meaningful dialogue about existential problems and resources between GPs (EMAP) and their cancer patients.

## Methods

The tool development was part of a larger mixed-methods quality improvement research project in Denmark having as overall aim to develop and evaluate a communication course that focuses on qualifying GPs’ communication with seriously ill patients about existential problems and resources. The tool described in this paper is both intended for communication training during the course and routine clinical care independently of course participation.

**Figure 1.**
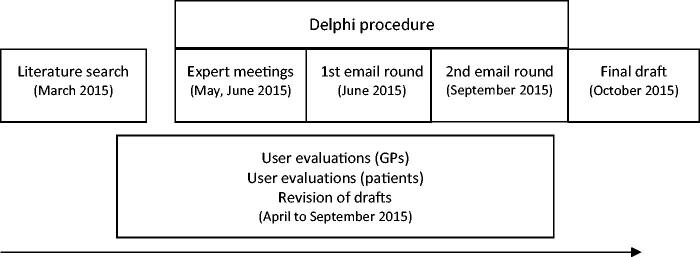
Developmental processes.

The development of the tool included several processes (Figure 1), some of which ran simultaneously.

### Literature search

To inform the drafting of the Danish general practice tool, we searched the literature for dialogue-oriented tools addressing patients’ existential problems, needs and resources. We searched PubMed MEDLINE and Scopus up to 2015 and used blocks of search terms closely related to ‘existential’ and ‘tool.’ In 2013, Luchetti et al., conducted a systematic review of tools and identified and provided an overview of dialogue-oriented tools in medical practice [10]. Up to 2015, the database search led to the identification of three additional tools [15–17]. These, together with the tools identified by Luchetti et al., were reviewed for clinical setting, structure, themes and questions. Tools were included if they were (1) developed for broad clinical usage, (2) semi or low-structured, (3) exploring existential themes, with (4) questions held in a religiously neutral language. The following guides were included: FICA [18], HOPE and the spiritual history [19,20], all of which had been developed by physicians for general clinical usage. All three tools incorporated the following core elements: assessment of patient’s coping, the kinds of support systems available to the patient, and any strongly held beliefs that might influence medical care. The sample of tools was presented to the experts in their original language (English) as preparation for the expert group meetings during which the overall intent, structure and content of the new tool would be discussed before pursuing the Delphi methodology. Furthermore, as will be described in the following section, a preliminary tool was drafted based on the findings of the literature search and presented to users during focus group interviews.

### User evaluations

To Increase the extent of user involvement, the preliminary drafts of the Danish tool were presented to samples of GPs and cancer patients separately during 13 focus group interviews conducted from April to September 2015. Two semi-structured interview guides were developed for each sample and GPs and patients were asked to critically evaluate the tool with respect to structure, content (missing themes, alternative wordings), and how the tool should be used. In addition to these evaluation questions, the interview guides also consisted of questions exploring GPs’ understanding and integration of the existential dimension and the patients’ perceptions and experiences of GP-provided existential care [21].

The GPs for seven focus groups (with 3–6 participants per group) were selected from two of the five regions in Denmark. The final sample consisted of 31 GPs (17 males and 14 females) between 36 and 68 years of age with practice experience ranging from three to 42 years. In total, 19 GPs were located in rural areas and 12 in urban areas.

The patients for six focus groups (with three-to-six participants per group) consisted of 24 individuals (16 women and eight men) between 30 and 75 years of age with mixed diagnoses (breast, prostate, colorectal, ovarian, uterine cancer, lymphoma, leukaemia and sarcoma). They were purposefully selected and recruited through patient organizations in The Southern Denmark Region. After each focus group, the first author (EAH) transcribed the recorded discussions (lasting between 60–75 minutes) in full. The data were analysed by EAH and NCH according to the core principles of a thematic analysis approach [22]. The analytic process has been described in detail elsewhere [21]. In between focus groups, the tool was adjusted such that the next focus group received an updated version based on previous feedback.

### Delphi procedure and expert panel

The essence of the Delphi method is that statements or items are qualified through sequential feedback rounds among a panel of experts until consensus is attained [23,24]. The panel was constituted to include multidisciplinary expertise relevant to the development of the tool. Other inclusion criteria were: nationally and internationally recognized senior faculty, with a PhD in health sciences, social sciences and/or humanities with scholarly productivity (including original research, peer-reviewed publications) within the subject of general practice/primary care, doctor–patient communication, cancer care (including palliative care) as well as spirituality and health. Fourteen experts were purposefully sampled and represented the following academic disciplines: medicine, nursing, psychology, sociology, and theology.

### Expert meetings

Two expert meetings took place in May and June 2015. The starting point for the expert meetings was the following open-ended points of discussion: (1) overall Intention of the tool, (2) structure, (3) content and (4) questions and formulations. The tool evaluated during focus groups was presented to the experts as a preliminary draft and compared with the international tools included through the literature search. Both meetings were moderated by EAH and tape-recorded. Sections of the audio that were responses to the four main questions above were transcribed verbatim and codes (recurrent ideas, themes, terms, phrases) were organized in a code list Box 1 that was emailed to the experts for verification.

Box 1. Code list deriving from analysis of expert meeting data.

- **Intent**

- Deepening of the doctor–patient encounter/relationship
- No specific agenda (e.g. problem solving, ‘fixing’) but exploration of patient’s lifeworld
- Showing non-strategic interest in the patient
- A tool that focuses more on the GP’s being than doing
- A source of inspiration for the GP
- Providing the GP with suggestions for structure, content and phrasing that might make him/her feel more ‘safe’ in the consultation (courage enhancement) so that he/she can focus on listening and
- Enhancing the GP’s capability to meet the other
- Enhancing of patient’s existential and spiritual well-being, reducing anxiety, increasing the empowerment of the patient
- Help to make the patient reach his/her goals (by referring to other specialists) and identify barriers to reaching these goals
- Part of a communication course in existential communication in which attitudes more than skills are being trained

- **Structure**

- Semi-structured with open-ended questions
- The tool should leave room for the GP’s own judgement and for improvisation.
- Division into themes and questions that map the structure of the tool:
- Introduction: Spotting of the patient, inviting him/her for an existential conversation and confirmation of the intent of the consultation should be mentioned in the introduction of the tool.
  - Focus on identification and assessment of patient’s problems/concerns/worries, and
  - patient’s resources/strengths/wishes and hopes.
  - Common action plan (future consultation with GP and/or other specialists) and verification of patient’s agreement in decision making/action plan.

- **Content**

- Existential themes and conditions: hope/hopelessness, meaning/meaninglessness, joy/anger, loneliness/connectivity, etc.
- The tool should give room for contemplating difficult feelings and conditions before focusing on resource optimization
- Talking about a patient’s faith, religion and/or spirituality (being potential tabooed topics) should be kept in a normalizing tone so as to facilitate disclosure and avoid feelings of shyness, shame or of being different

- **Wording**

- Avoidance of using complex concepts such as meaning, hope, etc. Instead, invite the patient to create his/her own wording and formulations in daily, spoken language possibly followed by the GP supplementing the wording with concepts, e.g. ‘I’m interested in how you are doing!’ ‘What is important to you?’ ‘What gives you peace of mind?’ ‘What prevents you from achieving your goals and wishes?’

### Delphi email rounds

The results of the discussions from the expert meetings informed the drafting of a 24-item tool that was mailed to the panellists (through SurveyXact).

In the first round, panellists were asked to rate the relevance of the 24 questionnaire items on a five-point Likert scale, ranging from 1 (= completely disagree) through 3 (= indifferent) to 5 (= completely agree). Experts were also given the opportunity to add free text comments and add additional or alternative proposals or wordings for all tool items. Two email-reminders were sent in each round. After the first round, the analyst (EAH) reviewed the data. The method of analysis was a content analysis including line-by-line coding of the free text provided by the panel members for the purpose of editing the tool to reflect both the textual and numerical feedback of the panel members [22].

During the second round, the panellists were presented with the anonymous ratings, written comments for each of the tool items of the first round, and asked to repeat the rating procedure of round one.

## Results

### User evaluations

*Structure*. Both GPs and patients appreciated that the tool was divided into key themes and key questions thus providing an insight into concrete existential problems and resources.

*Content.* GPs were concerned that, by asking questions about patients’ problems and concerns, they would run the risk of imposing problems on the patient. A broad opening question that did not imply existential distress was suggested together with questions identifying patient resources. GPs suggested that support from patient organizations and social workers be added to the listing of possible resources presented in Table 1. Patients suggested that nature outings and hobbies be added to the list of possible resources and that the list should include more resources of a spiritual than religious nature.

**Table 1.** Second Delphi round with the final themes, questions and level of endorsement.

Included items	Agreement (≥75%) among respondents (*n* = 14) *n* (%)
**EMAP—existential communication in general practice**	
This tool contains examples of themes, questions and sub-questions that might be addressed in a 30-min consultation during which the GP focuses on dialoguing about existential problems and resources with a patient. The GP invites a patient who, according to the GP, could benefit from such a dialogue. During the initial invitation, it might be useful to prepare the patient for the consultation by informing the patient about its focus on existential illness aspects.	13 (93)
**INTRODUCTION** I am very glad that you have agreed to participate in this conversation with me, so that we can see if we can identify something that I can help you with as your GP.	13 (93)
**IDENTIFICATION OF THE PATIENT’S PROBLEMS** (e.g. anxiety, anger, distress, hopelessness)	13 (93)
How are you? If the patient diverts away from the question or is only referring to physical symptoms, try to prompt him/her with the following questions:	13 (93)
What does it feel like to be you at the moment?	12 (86)
What thoughts/concerns/worries fill your mind right now?	13 (93)
Are you able to find peace of mind?	12 (86)
Having a serious illness may give rise to thoughts about an uncertain future, about whether one will get well again or why one has a serious illness (why me?). Have you had such thoughts?	13 (93)
Some people may experience feelings of anger (e.g. toward their physicians, their spouse and/or God), hopelessness or powerlessness. Is that something that you can recognize?	14 (100)
Are there any feelings that are particularly difficult for you to cope with? (e.g. self-reproach/sense of guilt/hopelessness).	14 (100)
Do you hope to return to or be able to perform previous everyday life activities?	13 (93)
Do you hope you could be more at peace and obtain a greater inner strength?	12 (86)
Do you have any particular hopes for the future? (If the patient cannot be cured, the question should focus on setting achievable goals for the future).	14 (100)
**IDENTIFICATION OF THE PATIENT’S RESOURCES**	14 (100)
Do you have something or somebody in your daily life that can support you?	14 (100)
What does this support help you achieve/feel? (e.g. meaning, comfort, courage, hope, care)	13 (93)
In the past, what has helped you through difficult times?	14 (100)
Do you think that this might help you in the situation that you are in right now?	14 (100)
For some people belief in something or somebody provides a sense of meaning and peace of mind—do you feel the same way? If yes:	14 (100)
Are there any activities that you can think of that give you peace of mind? (e.g. mindfulness, meditation, nature outings, physical activity, hobbies, prayer, attending church, reading of holy texts, etc.).	14 (100)
**CONCLUSION AND ACTION PLAN**	14 (100)
What do you think might help you in your current situation?	13 (93)
Are there any things that you can think of I can do to support you as your GP?	14 (100)
Would it be helpful to you if you could talk with somebody else (a psychologist, social worker, chaplain, imam)?	14 (100)
Would you like me to help you establish contact with e.g. a psychologist/chaplain/imam/social worker/patient organization?	14 (100)
Would you like to discuss these matters with me again at a future date?	14 (100)

*Recommended use.* Both GPs and patients recommended that the tool is used as a guide and source of inspiration and not as ‘yet another’ checklist. Patients stressed that the tool should primarily serve to assist the GP in seeing and listening to the patient.

### Expert meetings

A detailed overview of the results of the expert meetings is provided in Box 1. Regarding the overall intention of the tool, the experts agreed that it should facilitate GP–patient communication to empower patients toward awareness of existential problems and resources, facilitating action plans with the GP as a resource of support.

Regarding recommended structure and content, the experts agreed that the tool should serve as a semi-structured tool containing suggestions for existential themes concretized through open-ended questions. The experts suggested that the first theme should address the self-reported existential influence of the illness on the patient followed by another overall theme focusing on patient resources, values and strengths. Furthermore, it was considered important to thematize the patient’s goals, wishes and hopes for the future and to clarify whether the patient needs help for achieving this.

### Delphi survey

A response rate of 100% was obtained in both rounds. In the first round, 17 out of the 24 items under the seven themes received more than 75% expert consensus. Whether an item receiving ≥75% expert consensus should be included in the subsequent round was also made dependent on the written comments of the panel members that in most cases provided elaboration and clarification of the chosen response category leading to changes of questions and wordings.

Regarding the overall structure of the question tool, there were comments that recommended a more clear-cut division between questions that were meant to identify problems and those meant to identify resources (instead of mixing them across themes). It was suggested to structure the tool around four themes each containing suggestions for main questions as well as sub-questions: (1) introduction (with a suggestion for how the conversation might be initiated by the GP), (2) identification of problems (3) identification of resources, and (4) conclusion. This structure reflected the structure of the Calgary-Cambridge Guide; an evidence-based delineation of the basic communication skills that makes a difference in the communication with patients [25]. The drafting of the question tool for the second email round was revised accordingly (Table 1). Although consensus was achieved, the comments supplementing and explaining the ratings left room for further optimization of concrete wordings.

The EMAP acronym and mnemonic of the tool was developed (existential communication in general practice), indicating the intention of the tool: to provide a map of possible existential problems and resources that the GP and the patient can dialogue about and with this hopefully find points of reorientation in the patient’s illness situation.

## Discussion

This is the first north European study in which a multidisciplinary research team has worked together to develop a tool for GPs for communication with patients with cancer about existential problems and resources.

In mapping out the existential dimension related to cancer, the EMAP tool may help break down barriers related mainly to perceived lack of language and knowledge of the concepts involved. However, many other obstacles may be faced in the implementation process of the tool pertaining to the dominating healthcare culture of today. In a forthcoming publication about GPs’ self-perceived barriers for communication about existential issues [26], we show how the GPs experience and interpret individual barriers as being created and fostered within dominating biomedical and secular cultures characterized as ‘solution-focused’ and ‘faith-frightened.’ It is therefore acknowledged that the use of the EMAP tool will be qualified and its implementation advanced through continuing education that facilitates GPs’ reflection on how ‘macro,’ society-level dynamics influence the individual behaviour of GPs and their current practice culture. In relation hereto, it will be of equal importance to foster sensitivity to the GP’s values and beliefs, and how these might influence judgement regarding the patient’s existential needs. Moreover, knowledge of how to engender hope and how to turn patients’ needs into action plans, e.g., when referral to other care professionals is needed, is also an important educational step in advancing the implementation of the EMAP tool.

Future direction for research includes evaluation of the tool in different European contexts, development of courses based on the themes covered in the tool and the integration of the tool into these communication courses targeting GPs.

### Strengths and limitations

The strength of this study is that the final product, the EMAP tool, encompasses insights from all research processes. Thus, the EMAP tool is structured around the core tool elements identified through the literature search and revised according to feedback from patients, GPs and experts.

Both the length of the EMAP tool and the division into the identification of patients’ problems and resources could be considered a limitation. It must be stressed, however, that the tool is not intended for stringent survey style use, but as a comprehensive and flexible guide providing suggestions for how to address and dialogue about existential themes. Such dialogue represents a type of care that goes beyond the clinical impulse and imperative to act, to fix and to solve problems wherefore the EMAP tool is also an invitation to challenge the dominating bio-medical culture of Western European medicine.

Although cancer and general practice in Denmark have constituted our empirical starting point, the EMAP tool has been developed to outreach these boundaries in that it deals with broad and universal existential themes that play a central role to many individuals who suffer from serious or life-threatening illnesses or impairments [27].

## Conclusion

With a mixed method design, we developed the EMAP tool—a broadly framed, semi-structured tool to support GPs when communicating with patients with cancer about existential problems and resources. The tool contained suggestions for questions and sub-questions covering the existential dimension. Understanding the patient through dialogue about their concerns, beliefs and resources may benefit the patient by lowering existential distress and improve overall health and well-being. Sharing these sensitive issues in the GP–patient relationship is likely to increase both patient satisfaction and GP work satisfaction.
